# Mitigating HLA Disparity in AML Transplantation: Comparable Outcomes After Haploidentical and 9/10 Mismatched Unrelated Donor Transplantation With Treosulfan and PTCy

**DOI:** 10.1002/ajh.70483

**Published:** 2026-08-22

**Authors:** Daniele Avenoso, Mirko Farina, Michele Malagola, Yves Chalandon, Giorgia Saporiti, Annalisa Imovilli, Enrico Derenzini, Peter Remenyi, Massimo Martino, Maija Itala‐Remes, Renato Fanin, Didier Blaise, Desiree Kunadt, Arancha Bermudez Rodriguez, Franca Fagioli, Thomas Schroeder, Irene Cavattoni, Maud Ngoya, Mohamed Houhou, Mohamad Mohty, Eolia Brissot, Fabio Ciceri

**Affiliations:** ^1^ Unit of Blood Diseases and Bone Marrow Transplantation, Department of Clinical and Experimental Science University of Brescia, ASST Spedali Civili di Brescia Brescia Italy; ^2^ Service D'hematologie, Departement d'Oncologie, and Faculty of Medicine University of Geneva Geneva Switzerland; ^3^ BMT Unit, Hematology Division Fondazione IRCCS—Ca' Granda OMP Milano Italy; ^4^ Haematology Azienda USL‐IRCCS di Reggio Emilia Reggio Emilia Italy; ^5^ Oncohematology Division European Institute of Oncology, IRCCS Milan Italy; ^6^ Department of Health Sciences University of Milan Milan Italy; ^7^ Del‐Pesti Centrumkorhaz Budapest Hungary; ^8^ Grande Ospedale Metropolitano Bianchi Melacrino Morelli—Centro Unico Trapianti A. Neri Reggio Calabria Italy; ^9^ Turku University Hospital Turku Finland; ^10^ Azienda Sanitaria Universitaria Friuli Centrale Udine Italy; ^11^ Programme de Transplantation&Therapie Cellulaire Marseille France; ^12^ University Hospital TU Dresden Dresden Germany; ^13^ Hematology Department Hospital Universitario Marques Valdecilla‐IDIVAL Santander Spain; ^14^ Ospedale Infantile Regina Margherita, University of Turin Torino Italy; ^15^ University Hospital, Essen Essen Germany; ^16^ Hospital San Maurizio Bolzano Italy; ^17^ Department of Hematology EBMT, Sorbonne University, Hôpital Saint Antoine Paris France; ^18^ IRCCS San Raffaele Hospital Scientific Institute Milano Italy

## Abstract

Allogeneic hematopoietic stem cell transplantation (allo‐HSCT) is a potentially curative strategy for acute myeloid leukemia (AML), but the impact of HLA disparity in the era of posttransplant cyclophosphamide (PTCy) and reduced‐toxicity conditioning remains unclear. We performed an EBMT registry study including 275 adult AML patients in first complete remission undergoing first allo‐HSCT between 2012 and 2023 using a uniform platform of treosulfan‐based conditioning and PTCy. Among them, 206 received haploidentical grafts and 69 underwent 9/10 mismatched unrelated donor (MMUD) transplantation. Median follow‐up was 2.5 years. At 2 years, overall survival was 73.1% after haploidentical transplantation and 71.6% after MMUD transplantation (*p* = 0.96). Leukemia‐free survival at 1 year was 66.3% and 65.2%, respectively (*p* = 0.76). Relapse incidence (17.1% vs. 18.7%, *p* = 0.84) and nonrelapse mortality (16.6% vs. 16.1%, *p* = 0.86) were comparable between groups. Rates of Grade II–IV and Grade III–IV acute GVHD were similar, whereas chronic GVHD was higher after haploidentical transplantation (36.4% vs. 12.7%, *p* = 0.001), without differences in extensive forms. In multivariable analyses, donor type was not associated with overall survival, leukemia‐free survival, relapse incidence, or nonrelapse mortality. Increasing age adversely affected outcomes, while Karnofsky performance status ≥ 90 and reduced‐intensity conditioning were associated with improved survival. These findings suggest that, within a treosulfan–PTCy platform, major transplant outcomes are comparable between donor types, although haploidentical transplantation was associated with a higher incidence of chronic GVHD.

## 1 Introduction

Allogeneic hematopoietic stem cell transplantation (allo‐HSCT) remains the only curative option for many patients with acute myeloid leukemia (AML) [1, 2, 3], yet its success has historically depended on donor availability and the degree of HLA matching [4]. Fully matched donors have long been preferred due to the increased risks of graft‐versus‐host disease (GVHD) and non‐relapse mortality associated with HLA disparity, limiting access to transplantation for a substantial proportion of patients [5, 6, 7].

The introduction of post‐transplant cyclophosphamide (PTCy) has fundamentally changed this paradigm by enabling effective control of alloreactivity across donor types, significantly reducing both acute and chronic GVHD [8, 9]. In parallel, treosulfan‐based conditioning has emerged as a reduced‐toxicity platform that preserves antileukemic activity while minimizing regimen‐related toxicity, particularly in older or comorbid patients [10, 11, 12]. Whether HLA disparity continues to influence transplant outcomes is still unclear in the context of reduced toxicity conditioning regimes. Although outcomes with haploidentical and mismatched unrelated donors have improved in the PTCy era [13, 14, 15, 16], direct comparisons within a uniform platform combining reduced‐toxicity conditioning and PTCy as GVHD prophylaxis are scarce.

In this EBMT registry study, we evaluated adult AML patients in first complete remission (CR1) undergoing allo‐HSCT from either haploidentical or 9/10 mismatched unrelated donors, all treated with treosulfan‐based conditioning and PTCy. We aimed to determine whether donor type remains a clinically relevant determinant of outcome within this platform, or whether toxicity modulation and immune control have superseded HLA matching as the dominant drivers of transplant success.

## 2 Methods

### 2.1 Patient Selection

We included adult patients aged 18 years or older with a diagnosis of acute myeloid leukemia, excluding acute promyelocytic leukemia, who underwent a first allogeneic hematopoietic stem cell transplantation in first complete remission between January 2012 and December 2023. Eligibility was restricted to patients receiving treosulfan‐based conditioning and GVHD prophylaxis based on PTCy, administered in combination with institutional immunosuppressive agents. Only patients transplanted from either a haploidentical related donor or a 9/10 HLA‐mismatched unrelated donor were included. Patients receiving ex vivo T‐cell depletion or alternative GVHD prophylaxis strategies were excluded. Both peripheral blood and bone marrow grafts were allowed.

### 2.2 Definitions

Overall survival was defined as the time from transplantation to death from any cause, with surviving patients censored at last follow‐up. Leukemia‐free survival was defined as survival without evidence of relapse, with events including either relapse or death, whichever occurred first. Nonrelapse mortality was defined as death in the absence of prior relapse and was analyzed within a competing risks framework, considering relapse as the competing event. Relapse incidence was defined as the occurrence of hematologic relapse after transplantation, with death without relapse treated as a competing event.

Acute graft‐versus‐host disease was graded according to standard criteria, and the cumulative incidence of Grade II–IV and Grade III–IV acute GVHD by day 100 was assessed. Chronic GVHD was classified according to EBMT criteria, and both overall and extensive forms were evaluated. GVHD‐free, relapse‐free survival was defined as survival without Grade III–IV acute GVHD, severe chronic GVHD, relapse, or death.

### 2.3 Conditioning Regimen and GVHD Prophylaxis

All patients received fludarabine–treosulfan or fludarabine‐treosulfan‐thiotepa conditioning regimen followed by unmanipulated allogeneic HSCT. The cumulative treosulfan dose was selected according to patient characteristics and institutional practice. All patients had GVHD prophylaxis based on post‐transplant cyclophosphamide (PTCy—dose and days of administration were not available due to the retrospective and registry‐based study design). Additional immunosuppressive agents were administered according to donor type and individual center policy. The most commonly adopted regimens consisted of ciclosporine plus mycophenolate mofetil (MMF), whereas ciclosporine (CSA) alone, CSA/methotrexate (MTX), CSA/MTX/MMF, MTX alone, and other combinations were used only in a limited number of patients. The complete distribution of GVHD prophylaxis regimens is reported in Table 1. The use of anti‐thymocyte globulin (ATG) was left to the discretion of the treating center according to institutional practice and donor‐related considerations.

**TABLE 1:** Demographic characteristics of the study population.

		Total	Haplo	MMUD	Test *p*
		275	206	69	
Age at HSCT (years)	Median (range) [IQR]	61.82 (18.1–75.4) [52.3–68.2]	62.3 (18.1–75.4) [52.8–68.2]	60.3 (22.2–73) [51.1–67.4]	0.3915
Donor age	Median (range) [IQR] Missing	36.32 (18.5–206.5) [27.9–44.5] 25	37.9 (18.5–68.8) [30.2–46.1] 13	29.1 (20.4–206.5) [25.4–41] 12	0.0003
Year at HSCT	Median (range) [IQR]	2021 (2012–2023) [2019–2022]	2021 (2012–2023) [2019–2022]	2021 (2014–2023) [2020–2022]	0.0214
Time from diagnosis to HSCT (months)	Median (range) [IQR]	5.46 (2.2–26.9) [4.2–7.6]	5.6 (2.2–26.9) [4.2–7.6]	5.1 (2.6–24.1) [4.2–7.6]	0.8474
SEX	Female	116 (42.2)	92 (44.7)	24 (34.8)	0.1504
	Male	159 (57.8)	114 (55.3)	45 (65.2)	
Donor sex	Female	100 (36.4)	75 (36.4)	25 (36.2)	0.979
	Male	175 (63.6)	131 (63.6)	44 (63.8)	
Female to male donor	No	228 (82.9)	171 (83)	57 (82.6)	0.939
	Yes	47 (17.1)	35 (17)	12 (17.4)	
PATIENT CMV	Negative	41 (15.5)	27 (13.5)	14 (21.5)	0.1195
	Positive	224 (84.5)	173 (86.5)	51 (78.5)	
	Missing	10	6	4	
DONOR CMV	Negative	118 (43.2)	81 (39.7)	37 (53.6)	0.0437
	Positive	155 (56.8)	123 (60.3)	32 (46.4)	
	Missing	2	2	0	
Disease status	1st complete hematological remission (CR)	275 (100)	206 (100)	69 (100)	Not done
Karnofsky	< 90	49 (18.2)	37 (18.4)	12 (17.6)	0.8882
	≥ 90	220 (81.8)	164 (81.6)	56 (82.4)	
	Missing	6	5	1	
CELLSOURCE	BM	20 (7.3)	20 (9.7)	0 (0)	0.0072
	PB	255 (92.7)	186 (90.3)	69 (100)	
DONOR type	Haplo	206 (74.9)	206 (100)	0 (0)	< 0.0001
	9/10 UD	69 (25.1)	0 (0)	69 (100)	
Cytogenetic	Adverse	46 (17.5)	33 (16.8)	13 (19.7)	0.677
	Favorable	6 (2.3)	4 (2)	2 (3)	
	Intermediate	211 (80.2)	160 (81.2)	51 (77.3)	
	Missing	12	9	3	
Conditioning intensity	Myeloablative conditioning (MAC)	159 (58.5)	131 (63.9)	28 (41.8)	0.0014
	Reduced intensity conditioning (RIC)	113 (41.5)	74 (36.1)	39 (58.2)	
	Missing	3	1	2	
PTCy use	Yes	275 (100)	206 (100)	69 (100)	Not done
Use of ATG	ATG No ATG	36 (13.1) 239 (86.9)	26 (12.6) 180 (87.4)	10 (14.5) 59 (85.5)	0.69
Conditioning	Flu Thio Treo	77 (28)	69 (33.5)	8 (11.6)	0.0005
	Flu Treo	198 (72)	137 (66.5)	61 (88.4)	
	Missing	1	0	1	
GVHD prophylasis	CSA based CSA MMF based CSA MTX based CSA MTX MMF based MMF based MTX based Other Missing	2 (0.7) 76 (27.7) 3 (1.1) 2 (0.7) 187 (68.2) 1 (0.4) 3 (1.1) 1	2 (1) 60 (29.1) 3 (1.5) 1 (0.5) 139 (67.5) 0 (0) 1 (0.5) 9	0 (0) 16 (23.5) 0 (0) 1 (1.5) 48 (70.6) 1 (1.5) 2 (2.9) 1	Not done

*Note:* Continuous variables were compared using the Wilcoxon rank‐sum test and categorical variables using the chi‐squared or Fisher's exact test, as appropriate. “Missing” indicates unavailable data for the corresponding variable.

### 2.4 Statistical Analysis

Baseline characteristics were compared using appropriate statistical tests according to data type and distribution. Overall survival and leukemia‐free survival were estimated using the Kaplan–Meier method and compared between groups using the log‐rank test. Outcomes subject to competing risks, including relapse incidence, non‐relapse mortality, and GVHD, were analyzed using cumulative incidence functions and compared using Gray's test.

Multivariable analyses were performed using Cox proportional hazards regression models for overall and leukemia‐free survival, while Fine–Gray subdistribution hazard models were used for relapse incidence and nonrelapse mortality. Variables included in the multivariable models were selected based on clinical relevance and included age at transplantation, Karnofsky performance status, cytogenetic risk category, conditioning intensity, conditioning regimen (Flu/Treo versus Flu/Thio/Treo), and donor type. Hazard ratios with corresponding 95% confidence intervals were reported, and all tests were two‐sided with a significance threshold set at 0.05.

## 3 Results

### 3.1 Patient Characteristics

A total of 275 adult patients with acute myeloid leukemia in first complete remission were included in the analysis. Of these, 206 underwent haploidentical transplantation and 69 received a 9/10 HLA‐mismatched unrelated donor transplant. The median follow‐up for the entire cohort was 2.5 years and was comparable between the two donor groups, allowing a balanced assessment of intermediate‐term outcomes.

Baseline characteristics were overall well distributed between groups as shown in Table 1. Median age at transplantation was similar between haploidentical and MMUD recipients (62.3 vs. 60.3 years, *p* = 0.39), and no meaningful differences were observed in performance status or time from diagnosis to transplantation. By study design, all patients were transplanted in first complete remission, ensuring a homogeneous disease status at baseline.

Some imbalances were noted in transplant‐related variables. Differences in conditioning intensity and stem cell source reflected variations in clinical practice across donor platforms, with a higher proportion of reduced‐intensity conditioning and peripheral blood grafts in the MMUD group. Regarding GVHD prophylaxis, most patients received PTCy in combination with mycophenolate mofetil (68.2%) or ciclosporin plus mycophenolate mofetil (27.7%), while other immunosuppressive combinations accounted for less than 5% of cases (Table 1). These differences were accounted for in multivariable analyses.

### 3.2 Overall Survival

Overall survival was comparable between donor groups. At 2 years, OS was 73.1% in the haploidentical group and 71.6% in the MMUD group, with no statistically significant difference (*p* = 0.96—Figure 1A). Survival curves remained superimposable over time, indicating no clinically meaningful advantage of one donor platform over the other.

**FIGURE 1:**
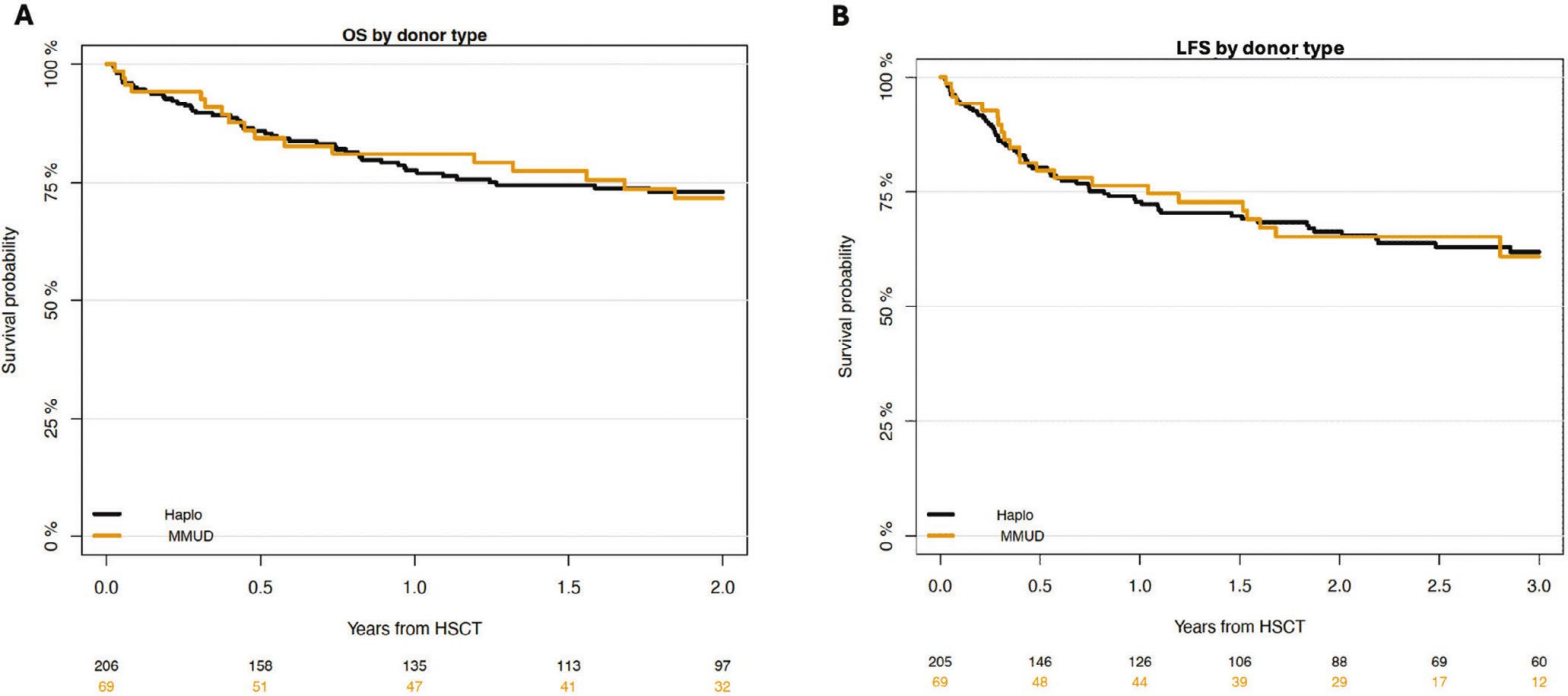
(A) Overall survival (OS); (B) Leukemia‐free survival (LFS). [Color figure can be viewed at wileyonlinelibrary.com]

These results indicate that, within this treatment platform, long‐term survival is not influenced by donor HLA matching.

### 3.3 Leukemia‐Free Survival

Leukemia‐free survival was comparable between donor types. At 1 year, LFS was 66.3% in the haploidentical group and 65.2% in the MMUD group, with no statistically significant difference (*p* = 0.76—Figure 1B). The Kaplan–Meier curves were closely overlapping throughout follow‐up, with no evidence of late divergence between the two donor platforms. The absence of separation between curves suggests that both relapse risk and non‐relapse mortality contribute similarly to outcomes in the two groups, supporting the consistency of the primary endpoint across donor types.

### 3.4 Relapse Incidence

The cumulative incidence of relapse was similar between the two groups. At 2 years, relapse incidence was 17.1% following haploidentical transplantation and 18.7% following MMUD transplantation, with no statistically significant difference (*p* = 0.84—Figure 2A). The cumulative incidence curves showed a parallel pattern over time, without evidence of increased relapse risk in either group. This finding suggests that the graft‐versus‐leukemia effect is preserved across both donor platforms within the context of treosulfan‐based conditioning and PTCy.

**FIGURE 2:**
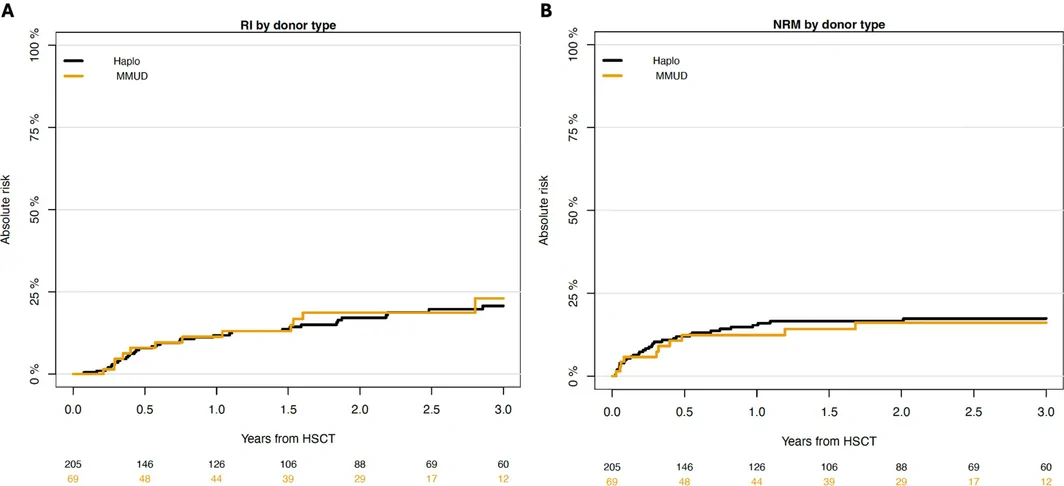
(A) Cumulative incidence of relapse; (B) nonrelapse mortality. [Color figure can be viewed at wileyonlinelibrary.com]

### 3.5 Nonrelapse Mortality

Nonrelapse mortality was also comparable between donor types. At 2 years, the cumulative incidence of NRM was 16.6% in the haploidentical group and 16.1% in the MMUD group (*p* = 0.86—Figure 2B). The temporal pattern of NRM was similar between groups, with no early or late excess mortality observed in either platform.

### 3.6 Graft‐Versus‐Host Disease

The incidence of acute GVHD did not differ between groups. By Day 100, Grade II–IV acute GVHD occurred in 24.7% of patients in the haploidentical group and 28.1% in the MMUD group (*p* = 0.70), while Grade III–IV acute GVHD was observed in 9.5% and 8.9% of patients, respectively (*p* = 0.99). In contrast, a significant difference emerged in the incidence of chronic GVHD. At 2 years, the cumulative incidence of chronic GVHD was substantially higher in the haploidentical group compared with the MMUD group (36.4% vs. 12.7%, *p* = 0.001—Figure 3A). However, the incidence of extensive chronic GVHD remained low and comparable between groups (5.0% vs. 6.0%, *p* = 0.95).

**FIGURE 3:**
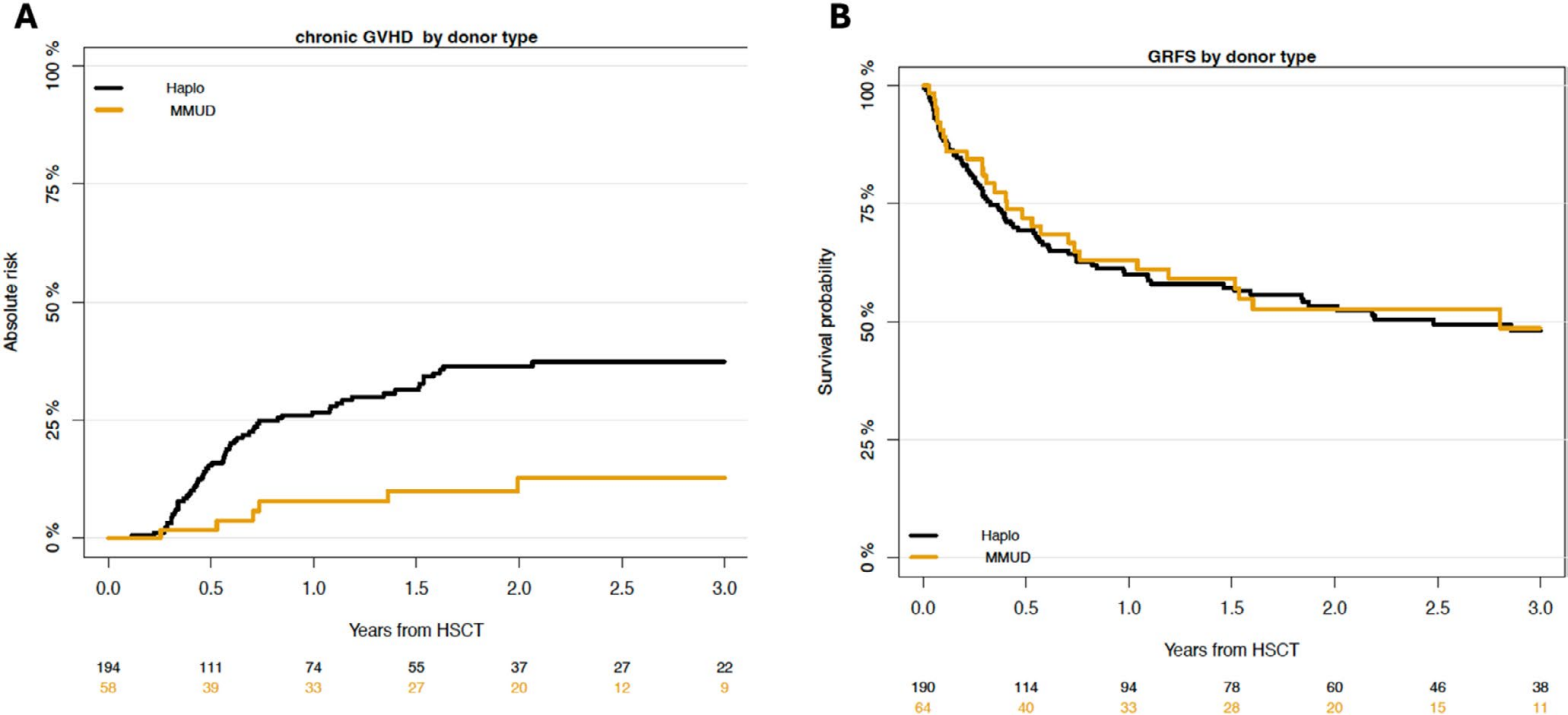
(A) Cumulative incidence of chronic GVHD; (B) GRFS. [Color figure can be viewed at wileyonlinelibrary.com]

### 3.7 GVHD‐Free, Relapse‐Free Survival

GVHD‐free, relapse‐free survival (GRFS) was comparable between the two donor groups (Figure 3B). At 2 years, GRFS was 52.6% and 53.3% for haploidentical and MMUD transplant, respectively (*p* = 0.63).

### 3.8 Multivariable Analysis

In multivariable analyses, donor type (MMUD vs. haploidentical) was not significantly associated with any of the major clinical outcomes, including leukemia‐free survival (HR 1.12, 95% CI 0.68–1.85; *p* = 0.66), overall survival (HR 1.35, 95% CI 0.78–2.32; *p* = 0.28), relapse incidence (HR 1.04, 95% CI 0.51–2.13; *p* = 0.91), or nonrelapse mortality (HR 1.28, 95% CI 0.62–2.61; *p* = 0.50). These findings confirm that donor HLA disparity does not independently influence transplant outcomes within this platform. In contrast, patient‐related variables retained prognostic significance. Increasing age was associated with inferior overall survival (HR 1.06 per 2‐year increase, 95% CI 1.01–1.11; *p* = 0.02) and higher non‐relapse mortality (HR 1.07, 95% CI 1.00–1.14; *p* = 0.04). A Karnofsky performance status ≥ 90 was independently associated with improved outcomes, including better leukemia‐free survival (HR 0.47, 95% CI 0.28–0.78; *p* = 0.004), overall survival (HR 0.55, 95% CI 0.32–0.95; *p* = 0.03), and lower nonrelapse mortality (HR 0.43, 95% CI 0.22–0.84; *p* = 0.01). Conditioning regimen (Flu/Treo vs. Flu/Thio/Treo) was not independently associated with LFS (HR 0.99, *p* = 0.98), OS (HR 0.79, *p* = 0.37), RI (HR 1.49, *p* = 0.27), or NRM (HR 0.66, *p* = 0.22). Conditioning intensity also influenced outcomes: reduced‐intensity conditioning was associated with improved leukemia‐free survival (HR 0.53, 95% CI 0.33–0.85; *p* = 0.008) and overall survival (HR 0.58, 95% CI 0.35–0.98; *p* = 0.04), with a trend toward lower nonrelapse mortality (HR 0.50, 95% CI 0.25–1.00; *p* = 0.05—Table 2).

**TABLE 2:** Multivariable analyses for leukemia‐free survival (LFS), overall survival (OS), relapse incidence (RI), and nonrelapse mortality (NRM).

		LFS		OS		RI		NRM		AGVH II–IV		AGVH III–IV		CGVH		CGVH extensive	
		HR (95% CI)	*p*	HR (95% CI)	*p*	HR (95% CI)	*p*	HR (95% CI)	*p*	HR (95% CI)	*p*	HR (95% CI)	*p*	HR (95% CI)	*p*	HR (95% CI)	*p*
Donor type	Haplo	1		1		1		1		1		1		1		1	
	MMUD	1.12 (0.68–1.85)	0.66	1.35 (0.78–2.32)	0.28	1.04 (0.51–2.13)	0.91	1.28 (0.62–2.61)	0.5	0.97 (0.54–1.75)	0.92	1.05 (0.39–2.84)	0.92	0.27 (0.1–0.73)	**0.009**	0.89 (0.23–3.43)	0.87
Year of HSCT (by 5 years)		1.26 (0.76–2.09)	0.36	1.42 (0.8–2.54)	0.24	1.04 (0.52–2.08)	0.9	1.56 (0.73–3.31)	0.25	2.07 (1.07–4)	**0.03**	0.85 (0.35–2.09)	0.72	0.6 (0.27–1.35)	0.22	5.25 (0.82–33.41)	0.08
Age at HSCT (by 2 years)		1.04 (1–1.08)	0.07	1.06 (1.01–1.11)	**0.02**	1.01 (0.96–1.07)	0.59	1.07 (1–1.14)	0.04	0.99 (0.95–1.03)	0.6	0.99 (0.92–1.06)	0.8	1.01 (0.95–1.07)	0.8	1 (0.89–1.11)	0.94
Female to male donor	No	1		1		1		1		1		1		1		1	
	Yes	0.9 (0.52–1.53)	0.69	0.93 (0.51–1.69)	0.81	0.74 (0.34–1.61)	0.45	1.12 (0.53–2.38)	0.77	2.18 (1.19–3.99)	**0.01**	1.22 (0.44–3.42)	0.7	1.29 (0.67–2.51)	0.45	2.32 (0.65–8.22)	0.19
CMV patient	Negative	1		1		1		1		1		1		1		1	
	Positive	1.14 (0.64–2.01)	0.66	1.02 (0.55–1.9)	0.94	1.21 (0.54–2.68)	0.64	1.1 (0.48–2.52)	0.82	1.15 (0.57–2.34)	0.69	1.27 (0.4–4)	0.68	0.66 (0.29–1.51)	0.32	0.85 (0.18–4.06)	0.84
Karnofsky	< 90	1		1		1		1		1		1		1		1	
	≥ 90	0.47 (0.28–0.78)	**0.004**	0.55 (0.32–0.95)	**0.03**	0.53 (0.24–1.18)	0.12	0.43 (0.22–0.84)	**0.01**	0.89 (0.46–1.71)	0.72	0.36 (0.15–0.87)	**0.02**	0.78 (0.31–1.99)	0.61	2.02 (0.24–17.02)	0.52
Cytogenetics	Adverse	1		1		1		1		1		1		1		1	
	Favorable/Intermediate	0.65 (0.4–1.06)	0.09	0.65 (0.38–1.1)	0.1	0.47 (0.25–0.88)	**0.02**	1 (0.44–2.26)	1	0.59 (0.33–1.05)	0.07	0.49 (0.2–1.21)	0.12	0.81 (0.39–1.68)	0.57	1.34 (0.29–6.27)	0.71
Regimen intensity	MAC	1		1		1		1		1		1		1		1	
	RIC	0.53 (0.33–0.85)	**0.008**	0.58 (0.35–0.98)	**0.04**	0.57 (0.3–1.1)	0.09	0.5 (0.25–1)	0.05	2.99 (1.7–5.27)	**0.001**	1.26 (0.53–3.02)	0.6	2.14 (1.01–4.54)	**0.048**	1.18 (0.31–4.41)	0.81
Conditioning	Flu Thio Treo	1		1		1		1		1		1		1		1	
	Flu Treo	0.99 (0.62–1.61)	0.98	0.79 (0.47–1.32)	0.37	1.49 (0.74–3.01)	0.27	0.66 (0.34–1.28)	0.22	1.04 (0.53–2.03)	0.92	1.23 (0.44–3.42)	0.69	1.01 (0.44–2.33)	0.99	0.76 (0.18–3.27)	0.71

*Note:* Hazard ratios (HR) and 95% confidence intervals (CI) were estimated using Cox proportional hazards models for OS and LFS and Fine–Gray subdistribution hazard models for RI and NRM. Reference categories were: Haploidentical donor, female‐to‐male donor = No, recipient CMV negative, Karnofsky < 90, adverse cytogenetics, MAC, and Flu/Thio/Treo conditioning. Age at HSCT and year of HSCT were modeled as continuous variables per 2‐year and 5‐year increase, respectively. Bold values indicate statistical significance (*p* < 0.05).

## 4 Discussion

In this EBMT registry study, we evaluated outcomes of adult patients with acute myeloid leukemia in first complete remission undergoing allogeneic transplantation with a uniform platform based on treosulfan conditioning and post‐transplant cyclophosphamide. Within this setting, we observed no significant differences between haploidentical and 9/10 mismatched unrelated donor transplantation across all major clinical endpoints, including leukemia‐free survival, overall survival, relapse incidence, and nonrelapse mortality. These findings were consistent in multivariable analyses, where donor type did not emerge as an independent determinant of outcome. Taken together, our results indicate that, in the context of reduced‐toxicity conditioning and effective immunomodulation, the historical disadvantage associated with HLA mismatch [7] seems to be substantially attenuated. This observation supports the concept that transplant outcome is increasingly driven by factors other than donor HLA matching, including patient‐related characteristics and the biological effects of the conditioning and GVHD prophylaxis platforms [17].

The biological plausibility of these findings lies in the combined effects of PTCy and treosulfan. Posttransplant cyclophosphamide selectively depletes proliferating alloreactive T cells while sparing regulatory and nonalloreactive subsets [18], thereby reshaping early immune reconstitution and reducing clinically significant alloreactivity. Additional modulation of immune reconstitution and GVHD biology through in vivo T‐cell depletion strategies has further emphasized the importance of immune control over simple HLA matching [19]. In parallel, treosulfan‐based conditioning provides effective myeloablation with reduced tissue damage and inflammatory signaling [20] compared with traditional regimens. The attenuation of tissue injury may limit the release of danger signals that amplify alloimmune responses, contributing to a lower functional impact of HLA disparity. Within this framework, donor mismatch may become less biologically relevant than in previous transplant eras.

Our findings are consistent with the growing body of literature demonstrating improved outcomes with alternative donor platforms in the PTCy era [21, 22]. However, most prior studies have included heterogeneous conditioning regimens and GVHD prophylaxis strategies, making it difficult to isolate the specific contribution of donor type. By focusing on a homogeneous platform combining treosulfan and PTCy, our study provides a more controlled comparison and suggests that, when toxicity and alloreactivity are effectively managed, differences between haploidentical and mismatched unrelated donors largely disappear.

An interesting and clinically relevant observation of our study is the higher incidence of chronic GVHD after haploidentical transplantation, in the absence of differences in extensive chronic GVHD. This pattern suggests that while chronic GVHD is more frequently diagnosed in the haploidentical setting, its severity may not be increased. These findings should also be interpreted in the context of the evolving epidemiology of chronic GVHD in the PTCy era, where changes in donor platforms, graft source, and immune prophylaxis strategies have modified both the incidence and clinical phenotype of cGVHD [23, 24]. Several factors may have contributed to this finding, including differences in donor platform, concomitant immunosuppression, use of serotherapy, patterns of immune reconstitution, and residual alloreactivity not fully suppressed by PTCy. Given that peripheral blood was used in all MMUD recipients and in the large majority of haploidentical recipients, graft source is unlikely to explain the higher incidence of chronic GVHD observed in the haploidentical group. Importantly, the lack of increase in severe chronic GVHD indicates that this signal should be interpreted cautiously and in the context of overall comparable survival outcomes.

Beyond donor type, our multivariable analyses confirmed the central role of patient‐related factors. Increasing age was associated with worse survival and higher nonrelapse mortality, while a preserved performance status was strongly associated with improved outcomes. These findings reinforce the concept that patient fitness and comorbidity burden remain key determinants of transplant success, even in the era of reduced‐toxicity conditioning.

The observation that reduced‐intensity conditioning was associated with favorable outcomes should be interpreted with caution, as it likely reflects patient selection rather than a direct causal effect. Nonetheless, it underscores the importance of tailoring conditioning intensity to individual patient characteristics within modern transplant strategies.

Our study has several limitations inherent to its retrospective design. Despite the use of a large registry dataset and a homogeneous treatment platform, residual confounding cannot be excluded, particularly given the observed differences in transplant‐related variables between groups. Information on molecular characteristics, measurable residual disease at transplantation, prior treatment history (including induction and consolidation strategies), comorbidities, and detailed immune reconstitution was not uniformly available in the EBMT registry and therefore could not be incorporated into the present analysis. In addition, center effects and variations in supportive care may have influenced outcomes. Also, the study period spanned more than a decade, during which haploidentical transplantation with PTCy became progressively established and center experience increased. Although year of transplantation was included in the multivariable analyses, temporal changes in transplant practice, supportive care, and institutional expertise may have contributed to the observed outcomes and should be considered when interpreting the results.

Despite these limitations, the strengths of our study include the relatively large cohort, the focus on a well‐defined disease status (CR1), and the use of a uniform conditioning and GVHD prophylaxis platform. These elements enhance the internal consistency of the analysis and allow for a more robust comparison between donor types than previously reported studies.

From a clinical perspective, our findings have direct implications for donor selection. In the setting of treosulfan‐based conditioning and PTCy, the choice between a haploidentical and a 9/10 mismatched unrelated donor may be guided primarily by donor availability, timing, and logistical considerations, rather than by concerns related to HLA disparity. Rapid access to transplantation remains a critical determinant of outcome in AML, and the availability of multiple effective donor options represents a major advantage in clinical practice.

In conclusion, in adult AML patients undergoing allogeneic transplantation in first complete remission, outcomes are comparable between haploidentical and 9/10 mismatched unrelated donors when using a treosulfan–PTCy platform. These results suggest that the impact of HLA mismatch is substantially mitigated in the context of modern transplant strategies, supporting a shift toward more flexible and patient‐centered approaches to donor selection.

## Funding

The authors have nothing to report.

## Ethics Statement

This study was conducted in accordance with the Declaration of Helsinki and approved by the institutional review procedures of the EBMT.

## Consent

All patients provided written informed consent for transplantation procedures and for anonymous use of clinical data for research purposes and for data collection and analysis according to EBMT standards and local regulatory requirements.

## Conflicts of Interest

The authors declare no conflicts of interest.

## Data Availability

The data that support the findings of this study are available on request from the corresponding author. The data are not publicly available due to privacy or ethical restrictions.
